# Effectiveness of care provided by an itinerant community caregiver in reducing the burden and violence of family caregivers of impaired elderly in Rio de Janeiro, Brazil: A randomized clinical trial

**DOI:** 10.1371/journal.pone.0309712

**Published:** 2024-12-05

**Authors:** Valéria Teresa Saraiva Lino, Nadia Cristina Pinheiro Rodrigues, Daniel Groisman, Soraya Atie, Luiz Antônio Bastos Camacho, Germana Perisse

**Affiliations:** 1 Primary Care Department- Germano Sinval Faria School Health Center, Sergio Arouca National School of Public Health- Oswaldo Cruz Foundation, Rio de Janeiro, Rio de Janeiro, Brazil; 2 Institute of Social Medicine, State University of Rio de Janeiro, Rio de Janeiro, Rio de Janeiro, Brazil; 3 Department of Epidemiology and Quantitative Methods in Health, Sergio Arouca National School of Public Health- Oswaldo Cruz Foundation- Rio de Janeiro, Rio de Janeiro, Rio de Janeiro, Brazil; 4 Joaquim Venâncio Polytechnic School, Oswaldo Cruz Foundation, Rio de Janeiro, Rio de Janeiro, Brazil; 5 Department for Dehospitalization Programs, Municipal Secretary of Health of Rio de Janeiro, Rio de Janeiro, Rio de Janeiro, Brazil; Utah State University, UNITED STATES OF AMERICA

## Abstract

**Introduction:**

The aging population and the rise in chronic diseases are linked to a higher number of elderly individuals with impairments. These individuals often depend on family caregivers for basic daily activities, which can impose a significant burden and increase the risk of violence against them.

**Objective:**

To assess the effectiveness of itinerant community caregivers (ICC) in reducing burden, depression and risk of violence among family caregivers of impaired elderly (FCIE), while also increasing their social support.

**Methods:**

Randomized controlled trial with 38 pairs of elderly people and their caregivers. For six months, twice a week, the ICC spent three hours with the elderly, completing tasks given by the FCIE. The primary outcomes were reduction of at least one level in the burden, and or in the risk of violence against the elderly. The secondary outcomes were a decrease in depressive symptoms and/or an increase in social support. Multiple log binomial regression models were used to assess the relationship between the predictors and the response variables.

**Results:**

In the FCIE group, most individuals providing care were women who spent over 16 hours each day in the task of caring for the impaired elderly, with most falling between the ages of 41 and 60. Over half of them were children of the elderly participants. In the intervention group, there was a significant decrease in the likelihood of violence against the elderly, with a 10-fold reduction. However, other endpoints did not present significant changes.

**Conclusion:**

The involvement of an ICC in the care of impaired elderly can contribute to reducing domestic violence by FCIE.

## Introduction

Approximately 200 million individuals around the world have significant functional dependence, requiring help to perform daily activities. Between the years 1990 and 2017, the total number of years lived with disability increased by more than 50% globally [1]. This situation shows a growing trend, due to population aging and the increase in the occurrence of chronic diseases, with a higher concentration of impaired elderly among those over 80 years old, the fastest-growing age group. In Brazil, the prevalence of dependence in performing basic activities of daily living ranged from 6 to 7% between 1998 and 2008, increasing to 15.5% in 2014 [2, 3]. Since then, the reality hasn’t changed, indicating that it is urgent to know the impact of dependence and the need for caregivers to operationalize public policies consistent with the country’s reality.

Caring for elderly individuals, especially those with chronic illnesses or disabilities, is a demanding and challenging role that can significantly impact the physical, emotional, and mental well-being of caregivers. Caregiver burden, referring to the physical, emotional, and financial strain experienced by those who care for elderly individuals, is common among this group [4].

An informal support system has taken on the responsibility of providing care, with middle-aged women primarily serving as family caregivers of impaired elderly (FCIE). The low level of schooling, the prolonged periods dedicated to this activity, and the lack of ability to the necessary care characterize the universe of FCIEs [5]. Although there are positive aspects of care, such as self-affirmation, a sense of satisfaction, and joy, caregivers may present depression, anxiety, insomnia, weight loss, and decreased quality of life [5]. They can experience pain and disability, which can respond to physical therapy programs [6, 7]. Also, caregivers who assist with a greater number of ADL/IADL activities, health management tasks, health systems logistics, and those using respite care are more likely to experience burden [8]. All these factors contribute to the emotional burden experienced in everyday life and negatively impact caregiving.

When a family caregiver looks after dependent elderly individuals, the resulting caregiver burden can heighten the risk of violence against the elderly which involves the potential for physical, emotional, or psychological harm inflicted by caregivers on elderly individuals [9, 10]. Globally, violence against the elderly has reached epidemic proportions, becoming a significant cause of morbidity and mortality, resulting in high individual and collective costs [11]. Underreporting worldwide leads to inaccuracies in statistics, and even in developed countries, it is estimated that for every reported case, there are several others unreported [12]. Elder abuse refers to an intentional act (single or repeated) or omission of care that causes harm or distress and occurs in any relationship where there is an expectation of trust. It causes harm or serious risk of harm to an older adult or deprives an older adult of basic needs [13]. The most common types of violence against the elderly population are physical, psychological, sexual, financial, and neglect. The latter, though common, is the most difficult to characterize [12]. Living alone with the perpetrator and having functional impairments are, among other factors, known risk factors for elder mistreatment [11]. Contributing factors include high levels of stress, lack of coping resources, poor caregiver mental health, and inadequate support systems. As a result, violence can have severe physical and psychological consequences for the elderly, including injury, trauma, and an increased risk of mortality [14].

Social support is important to alleviate the tension associated with caregiving [15, 16]. This refers to an individual’s satisfaction with their network of relationships, including expressions of affection, empathy, access to advice, help in times of need, and the availability of people to socialize with [17]. Without social support, the burden of caregiving can be doubled or tripled, with the most commonly reported need being assistance in providing care [18].

There is a significant gap in the evaluation of burden and violence faced by caregivers of the elderly by clinicians. Caregiver burden is often overlooked in medical consultations, resulting in inadequate support for those fulfilling this crucial role [16]. Furthermore, the identification and intervention in cases of violence against caregivers are rarely addressed, leaving many without the necessary help. There is an urgent need to incorporate effective assessment and intervention strategies into clinical management to mitigate the burden and violence faced by caregivers, thus ensuring a better quality of life for both the caregivers and the elderly under their care.

In Brazil, that gap is even more pronounced due to the scarcity of public services for impaired elderly individuals and the limited initiatives involving the employment of formal caregivers as part of public policies [19]. This research evaluates the effectiveness of the care provided by an itinerant community caregiver (ICC) in relieving FCIE burden and family violence by the caregiver against the elderly.

## Materials and methods

The recruitment began on February 4, 2019 and ended on April 30, 2019. The study was registered in the Brazilian Registry of Clinical Trials (REBEC) as RBR-4ytnzx. The CONSORT reporting guidelines was used for reporting the study [20]. A randomized controlled parallel clinical trial was conducted in Manguinhos, an area characterized by severe socio-environmental vulnerability situated in the northern region of Rio de Janeiro city. The study involved the participation of both primary care units dedicated to providing healthcare services to the local population. The six-month intervention featured care administered by an ICC, whereas the control group received instructions on caring for impaired elderly individuals during a single visit from a social worker. The eligibility criteria for participants were: elderly people (aged 65 or over) with three or more disabilities according to the Brazilian version Independence in Activities of Daily Living Index (Katz Index), accompanied by family caregivers. It contains six basic activities of daily living (ADL) related to self-care, and those who needed assistance with only one or two ADL were excluded due to their low level of dependence [21]. Elderly individuals without a family caregiver providing daily care were also excluded. Elderly people and their caregivers participated in the study in pairs. Sample size was calculated considering α = 5%, β = 20%, ratio between groups = 1, relative risk = 6, totaling 76 individuals. As described below, although 81 pairs were randomized, only 48 actually participated in the study.

In the records of the primary care units, we identified 129 eligible pairs. Of those, 81 accepted to participate and were randomly assigned. Participants were assigned to one of the study groups through a list of computer-generated random numbers, in blocks defined by a statistician not directly involved in the fieldwork and data analysis. There was no blinding of participants and professionals regarding the type of intervention allocated to participants. A member of the research team carried out the initial interviews of all participants at their homes. Eight ICC were selected from the local community, requiring them to have completed a professional caregiver training course. They cared for an elderly person for three hours, twice a week, performing tasks listed by the family member together with the research team. All ICC were supervised by the research team with weekly meetings and received payment for the work performed in the study.

The total study period lasted seven months, including a final evaluation conducted one month after the six-month interventions. The following aspects were evaluated in the FCIE: a) burden, with the Zarit Burden Interview Scale (ZBI)- It assesses the impact of mental and physical illnesses, providing the level of emotional burden on caregivers. Comprising 22 questions with five possible responses, indicating the frequency with which the individual feels overwhelmed in different situations. For each alternative, the score ranges from 0 (never) to 4 (almost always). In this study, three levels of burden were considered: absent or mild (less than 21); moderate (21–40); and severe (above 40 points). [22]; b) depression, with the Patient Health Questionnaire-9 (PHQ-9), to screen depression at the primary care level. It consists of nine questions that assess the presence of depression symptoms. The frequency of each symptom in the last two weeks is evaluated on a Likert scale from 0 to 3, corresponding to the responses "not at all," "several days," "more than half the days," and "nearly every day," respectively. The cutoff point for depression is ≥ 9 [23]; c) social support, with the Medical Outcomes Study Social Support Scale (MOS)- It consists of 19 questions covering the five dimensions of social support (affectionate, emotional, material, informational, and positive social interaction). The score is defined based on the frequency of support perceived by the individual in each dimension, ranging from zero (never) to four (always). In this study, unsatisfactory social support (USS) was considered when more than 50% of items in each dimension had responses of zero (never), one (rarely), or two (sometimes), meaning the total score must be less than 46 points [24]; d) increased risk of violence by FCIE with the Caregiver Abuse Screen (CASE)- The instrument contains eight items and is easy to administer. The response categories are in a dichotomized form, each capable of receiving zero or one point. Affirmative responses indicate the possibility of violence risk. In the original study, increased risk was identified starting from four points. Thus, in the absence of local studies validating other cutoff points, we opted to define increased risk of violence when at least 50% of the questions were affirmative, which equated to a score of four or more [25, 26]. The primary outcomes were reduction of at least one level in the ZBI and/or in the risk of violence against the elderly. The secondary outcomes were a decrease in depressive symptoms and/or an increase in social support. The final assessment was conducted by an interviewer who remained unaware of the specific intervention group to which the participants had been allocated. The study protocol and the dataset can be accessed in the Supporting information (S1 Protocol and S1 File).

A descriptive analysis of the distribution of FCIE was carried out in each intervention group according to gender, age group, education, depression, social support and burden. Proportions of burden reduction in each group, difference between proportions, as well as the 95% CI and the p-value (α = 0.05) of the difference between proportions were calculated. Then, the analysis was carried out according to the per-protocol, that is, only included those who fully received the intervention for which they were originally allocated. The burden reduction ratios were compared. Multiple log-binomial regression models were used to assess the relationship between the predictors (intervention group, male, married, years of care, hours of the day spent with the elderly), and the response variables (burden reduction, violence reduction against the elderly, depression reduction, and social support increase). Only for the model with the response variable "depression reduction", the predictor "male" was not included in the adjusted model. In all analyses, a significance level of 0.05 was used. To investigate multicollinearity, the Variance Inflation Factor (VIF) was calculated. The models were also tested for the presence of interaction (sex vs. hours with the elderly person; sex vs. marital status, sex vs. years of study; marital status vs. years of study; marital status vs. hours with the elderly person).

The public domain program R-project 4.3.3 was used. All participants signed the written informed and voluntary consent form and the study was approved by the National Public Health School Research Ethics Committee (number 45595215.4.0000.5240) (S1 Fig). Important changes to methods after trial commencement were not anticipated.

## Results

**Fig 1:**
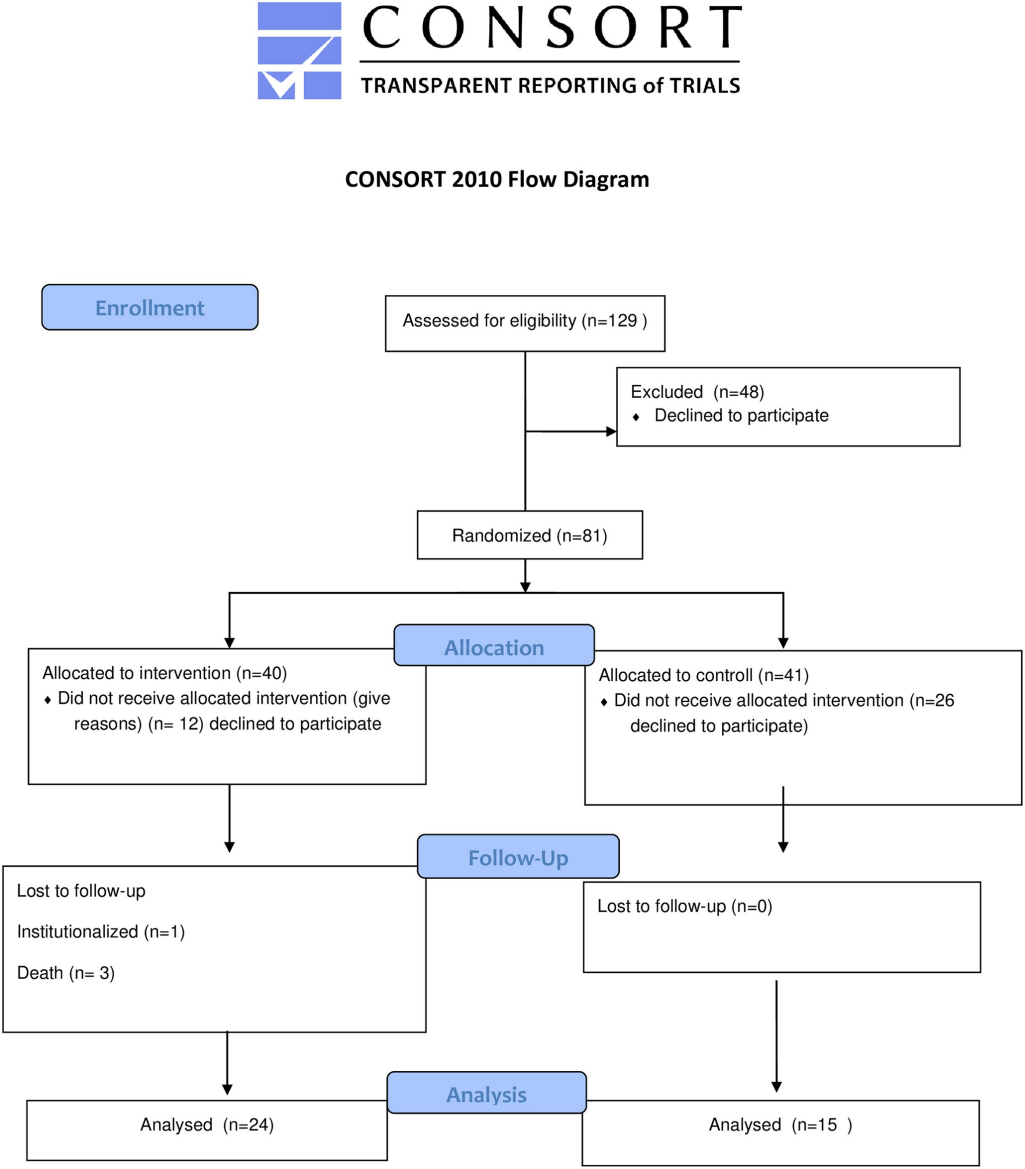
CONSORT flow diagram.

The visits were conducted from June to December 2019. Out of the 81 pairs that were randomized, 51 started the study. During the study period, three elderly participants in the intervention group passed away due to complications from pre-existing conditions. Ultimately, only 48 participants completed the study. Most participants who declined to participate cited distrust towards the offer of a free home service, difficulty receiving a stranger at home, or the belief that partial care would not be effective in alleviating their burden. The CONSORT flow diagram provides information about the enrolment, allocation, follow-up, and analysis of patients involved in the RCT (Fig 1).

It’s interesting to note that the majority of FCIEs were women, with most falling between the ages of 41 and 60. Additionally, over half of them were the children of the elderly participants. In a significant portion of cases, caregivers spent more than 16 hours providing care. Table 1 presents sociodemographic data of FCIEs, degree of kinship, and time spent on caregiving daily.

**Table 1:** Sociodemographic data of family caregivers of the impaired elderly, degree of kinship, and time spent on caregiving daily.

		FCIE	
		Intervention (n = 30)	Control (n = 18)
Gender (%)	Female	28 (68.3)	13 (31.7)
	Male	2 (28.6)	5 (71.4)
Age (years) (%)	≤ 40	3 (50.0)	3 (50.0)
	41–60	18 (60.0)	12 (40.0)
	>60	9 (75.0)	3 (25.0)
Education (years) (%)	≤ 5	9 (60.0)	6 (40.0)
	6–10	19 (70.4)	8 (29.6)
	>10	2 (33.3)	4 (66.7)
Degree of relationship (%)	Spouse	2 (66.7)	1 (33.3)
	Child	19 (59.4)	13 (40.6)
	Other	9 (69.2)	4 (30.8)
Daily tasks (workload) (%)	≤ 8h	4 (50.0)	4 (50.0)
	9-16h	5 (45.5)	6 (54.5)
	> 16h	9 (31.0)	20 (69.0)
Marital status (%)	Single	11 (61.1)	7 (38.9)
	Divorced	3 (60.0)	2 (40.0)
	Married	11 (55.0)	9 (45.0)
	Widow	5 (100.0)	0 (0.0)

A considerable number of FCIEs experienced moderate to high levels of burden, depression, and unsatisfactory social support, while almost half of this group presented an increased risk of violence (Table 2).

**Table 2:** Levels of burden, depression, unsatisfactory social support and risk of violence in family caregivers of the impaired elderly.

		Pre- intervention Groups:		Post- intervention Groups:	
		Intervention	Control	Intervention	Control
Burden (ZBI) (%)	No	9 (69.2)	4 (30.8)	4 (50.0)	4 (50.0)
	Yes	21 (60.0)	14 (40.0)	26 (65.0)	14 (35.0)
PHQ-9 (%)	No depression	14 (77.8)	4 (22.2)	10 (71.4)	4 (28.6)
	With depression	16 (53.3)	14 (46.7)	20 (58.8)	14 (41.2)
CASE (risk violence) (%)	No risk	19 (70.4)	8 (29.6)	13 (81.2)	3 (18.8)
	With risk	11 (52.4)	10 (47.6)	17 (53.1)	15 (46.9)
Social Support (%)					
	Satisfactory	15 (68.2)	7 (31.8)	20 (74.1)	7 (25.9)
	Unsatisfactory	15 (57.7)	11 (42.3)	10 (47.6)	11 (52.4)

CASE- *Caregiver Abuse Screen;* FCIE- Family caregivers of impaired elderly; PHQ-9- Patient Health Questionnaire-9; ZBI- Zarit Burden Interview

The outcomes analysis revealed a statistically significant difference only for the reduction of violence (p = 0.02). The group that received the ICC was 10 times more likely to have reduced violence when compared to the group that did not receive the intervention (Table 3). The same result was not observed regarding the reduction of burden and depression, or the increase in social support for caregivers.

**Table 3:** Adjusted effect of the predictors versus reduction of burden, risk of violence, depression, and increase in social support of family caregivers of impaired elderly.

	Burden reduction	Violence reduction	Depression reduction	Increased social support
**RR (CI 95%)**				
**Intervention (ICC)**	1.11 (0.49–2.56)^NS^	10.54 (1.36–81.87)*	1.08 (0.39–3.01)^NS^	0.82 (0.21–3.21)^NS^
**Male**	0.40 (0.05–3.05)^NS^	1.37 (0.51–3.69)^NS^		1.43 (0.22–9.26)^NS^
**Married**	0.37 (0.13–1.10)^•^	2.77 (1.34–5.73)**	0.95 (0.35–2.58)^NS^	0.60 (0.16–2.28)^NS^
**Years of Care**	0.93 (0.75–1.16)^NS^	1.20 (0.99–1.42)*	0.88 (0.74–1.04)^NS^	1.01 (0.82–1.24)^NS^
**Nº hours of the day spent with the elderly**	0.90 (0.84–0.96)*	1.06 (0.99–1.12)^•^	1.01 (0.93–1.10)^NS^	1.12 (1.01–1.24)*

ICC- Itinerant community caregiver; RR- relative risk (log binomial regression model); CI = Confidence Interval;

*** = p-value < 0.0001,

** = p-value < 0.001,

* = p-value < 0.01,

^•^ = p-value < 0.1

Multiple log-binomial regression models were used to assess the relationship between the predictors (intervention group, male, married, years of care, hours of the day spent with the elderly), and the response variables (burden reduction, violence reduction against the elderly, depression reduction, and social support increase). Only for the model with the response variable "depression reduction", the predictor "male" was not included in the adjusted model. In all analyses, a significance level of 0.05 was used.

Marital status influenced the likelihood of experiencing burden and violence. Divorced FCIEs were 3.58 times more likely to experience a reduction in burden compared to their married counterparts. Conversely, single FCIEs had a 57% lower probability of reducing violence than those who were married (Table 3).

The presence of interaction was tested (sex vs. hours with the elderly person; sex vs. marital status, sex vs. years of study; marital status vs. years of study; marital status vs. hours with the elderly person) and none of the interactions tested were significant. The presence of collinearity was investigated. Based on the estimated VIF values for the Adjusted Logistic model, all values are close to 1. This suggests that collinearity between the independent variables in your model is low.

## Discussion

This research assessed the impact of having a community caregiver assist in caring for impaired elderly for six hours per week over six months. Previous studies have found that those who require daily care often put a heavy burden on family caregivers, particularly women who make up the greater majority of FCIEs. This reinforces the evidence that in most Western societies, women, especially middle-aged women, are naturally expected to take on the responsibility of caring for their dependent relatives [2].

Moderate to high levels of burden were reported by 72.9% of the FCIEs before the intervention, a much higher rate than the 59.8% previously observed in the same community [18]. This prevalence is consistent with other studies in Brazil. Nardi et al. found that 75.2% of FCIEs in the southern part of the country reported moderate to severe burden, while Loureiro et al indicated that this rate was 84.6% in the northeastern region [27, 28].

Caregiver burden is a universal issue that impacts the health and quality of life of individuals across various cultures and countries. A World Health Organization survey involving 19 countries with different socioeconomic statuses found that nearly 40% of the 43,732 family caregivers interviewed reported experiencing burden. Additionally, the financial burden was more than twice as high in poorer countries compared to wealthier ones [15]. Despite the established link between caregiver stress and elderly dependence, this study found that the intervention with ICC did not result in a statistically significant reduction in burden, likely due to the small sample size.

It is no coincidence that most family caregivers spent over nine hours a day providing care, with nearly 60% dedicating more than 16 hours to this task. This substantial time commitment is a significant indicator of the burden caregivers face and is a well-established predictor of depression and stress among those caring for individuals with dementia or cancer [5].

Violence against the elderly is a recurring and often fatal phenomenon, resulting in high costs to the healthcare system. Elderly individuals who experience abuse are more likely to die within the first years following the traumatic event [29]. In this RCT, the risk of violence by FCIEs increased by 10.1% compared to a study conducted in the same community six years earlier [18]. However, the group that received the ICC intervention was 10 times more likely to experience a reduction in violence compared to the group that did not receive the intervention (Table 3). Pillemer et al. conducted a literature review on elder abuse, examining risk factors and prevention strategies based on the strength of the evidence. The authors found that disability, poor physical health, and cognitive impairment were strongly associated with violence against elderly individuals, whereas social support interventions proved to be protective. They suggested that caregiver interventions are a promising approach to prevention [30]. Despite the intervention not directly reducing the burden of caregiving, it’s conceivable that the care provided by the ICC in our study, even for a few hours a week, provided the essential social support to alleviate that burden significantly, thereby remarkably diminishing the risk of elder abuse.

In a study analyzing the frequency of repeated violence against elderly individuals between 2011 and 2018, Pampolim & Costa Leite suggested that signs of caregiver burden should be investigated to prevent violence [31]. Incorporating a straightforward and user-friendly tool such as the CASE into the regular practice of services catering to impaired elderly individuals may offer a promising avenue to illuminate abuse perpetrated by FCIEs.

In 2003, in Brazil, the "Statute of the Elderly" law was put in place. It represented a legal framework for the protection of the Brazilian elderly population, by regulating the rights of the elderly and establishing duties and punitive measures. According to the legislation, the well-being of the elderly is a shared responsibility of families, communities, society, and the government [32]. Despite the law, the inequality in access to health services and lack of support for poor families may create a form of structural violence in our environment. This could make it difficult to provide adequate care for dependent elderly individuals and could lead to a disproportionate level of violence associated with caregiver burden.

Although social support is known to alleviate the burden associated with caring for impaired elderly individuals, the intervention in this study did not alleviate the burden of FCIE [15]. This contrasts with other studies that have found a correlation between low social support and FCIE burden, as well as a previous study conducted in the same community [18, 33]. It is possible that the sample size may have affected the lack of association, or that a dose-response effect may be at play.

This study found that being divorced had a protective effect against caregiver burden. However, these findings should be approached with caution as they have not been consistently supported by previous studies. Other factors such as the need for assistance in care and the time dedicated to caregiving have been associated with an increased risk of burden [33–35].

This study carries several strengths, such as being a randomized clinical trial. This reduces the risk of sample selection bias and strengthens the results, showing a significant reduction in violence against impaired elderly when a community caregiver is involved. The sample used in this study was also population-based, and instruments adapted to our culture were employed, making it possible to compare with similar studies conducted in other communities. However, the loss of participants resulted in a decrease in sample power, which might impact the assessment of burden reduction from the intervention. A larger sample size would allow for more inferences. Moreover, it was not feasible to test the intervention with different groups receiving the ICC for over six hours a week to evaluate the dose-response effect, where a decrease in burden is proportional to the increase in work hours on the ICC. Finally, considering the study sample, it is not possible to generalize the results.

## Conclusions

This study demonstrated the effectiveness of a strategy aimed at reducing the risk of violence against elderly individuals who are dependent on others for care in poor communities. The approach involves assigning community caregivers to the homes of these individuals. This reduces the incidence of family violence and enhances the health system’s overall efficiency through the presence of these professionals.

To fully evaluate the strategy, testing it among other demographic groups is important. Conducting a cost-effectiveness assessment and analyzing the dose-response effect by varying the caregivers’ work schedules throughout the week will provide valuable insights for managers to assess the impact of the strategy.

## Supporting information

S1 Protocol Protocol study. (DOC)

S1 File Dataset. (CSV)

S1 Fig (PDF)
