# Supplementary material for: Effectiveness of care provided by an itinerant community caregiver in reducing the burden and violence of family caregivers of impaired elderly in Rio de Janeiro, Brazil: A randomized clinical trial
Source: PLoS One. 2024 Dec 5;19(12):e0309712. doi: 10.1371/journal.pone.0309712 (PMC11620672; doi:10.1371/journal.pone.0309712)
Supplement: S1 Protocol — (DOC) [file pone.0309712.s001.doc]

**Research Project**

Effectiveness of care provided by an itinerant professional caregiver in reducing the burden of family caregivers of dependent elderly people: a randomized clinical trial

Research Project

1. Valéria Teresa Saraiva Lino- médica- Escola Nacional de Saúde Pública Sergio Arouca/ Centro de Saúde Escola Germano Sinval Faria
2. Nádia Cristina Pinheiro Rodrigues- epidemiologista- Escola Nacional de Saúde Pública Sergio Arouca/ Centro de Saúde Escola Germano Sinval Faria
3. Soraya Athie- assistente social- trabalhadora autônoma
4. Luiz Antônio Bastos Camacho- epidemiologista- Escola Nacional de Saúde Pública Sergio Arouca/ departamento e Epidemiologia e Métodos Quantitativos em Saúde
5. Daniel Groisman- psicólogo- Escola Politécnica Joaquim Venâncio
6. Germana Périssé- médica- Gerência do Programa de Saúde do Idoso da SMSDC-RJ

1. INTRODUCTION

Approximately 200 million individuals around the world have significant functional dependence, requiring assistance to perform daily activities, a situation with an increasing trend due to population aging and the increased prevalence of chronic diseases. The highest concentration of dependent elderly people occurs among those over 80 years of age, the fastest growing age group1. This trend has also been observed in Brazil, whose dependency rate for carrying out basic activities of daily living was around 6 to 7% between 1998 and 20082. But, despite the knowledge of increasing levels of disability, there is still We are not able to measure the impact on the health system, both of dependence and the need for caregivers for dependent elderly people. Recognizing the role of the caregiver and making estimates about this population in our country is a necessity to be considered in the planning of public policies3.

The role of caregiver has been provided by an informal support system that includes friends, neighbors, but, mainly, relatives, who are family caregivers of dependent elderly people (CFID)4. Although there are positive aspects of caring, such as self-affirmation, a sense of satisfaction and joy5, this task often leads to depression, anxiety6, insomnia, weight loss and decreased quality of life7.

Middle-aged women predominate in the role of caregiver, although older women also assume this role. The low level of education, the prolonged periods dedicated to care and the lack of rotation in the task characterize the universe of family caregivers, with evidence that they have little control over health problems and also the necessary care required by the elderly6. These factors, combined with the absence of home services and cohabitation with dependent elderly people, contribute to the emotional stress experienced in daily life, but above all, the main source of burden is the degree of disability of the individual receiving care8.

In Brazil, it is estimated that almost 4 million adults devote 40 hours a week to caring for dependent family members9 and that the proportion between dependent elderly people and caregivers increased from 1:1 to 2:1 between the years 2000 and 201510. If the Care provided to just one individual with disability already imposes a burden on the caregiver; the prospect of doubling the number of dependent elderly people places an even greater burden on these family members.

The tension associated with the task of caring for dependent elderly people can be alleviated by social support (AS)11. This concept is related to the individual's satisfaction with their network of relationships, consisting of dimensions that concern expressions of affection (a. affective), empathy (a. emotional), access to advice (a. information), assistance in case of need (material support) and availability of people with whom to enjoy leisure (positive social interaction)12. Offering material and emotional support to the caregiver alleviates the burden and improves the conditions for caring for the frail elderly13. On the other hand, the absence of AS for spouses who take care of individuals with dementia is decisive for the persistence of symptoms of stress and depression for up to three years after the death of the dependent elderly person14.

Support groups for caregivers of dementia patients are beneficial for reducing depressive symptoms and improving the psychological well-being of caregivers. However, with regard to overload, the effect of this support is reduced, demonstrating the need for another type of support to deal with overload more effectively. Educational interventions are more effective than psychoeducational interventions, suggesting that the acquisition of skills to provide care, deal with legal issues and promote self-adjustment to the disease allows the caregiver to identify resources to deal with the burden imposed by dementia15.

It is necessary to carry out interventions capable of reducing the CFID burden, based on the reality of each region. Home care is a component of the care chain that aims to provide comprehensiveness, one of the objectives of primary care in Brazil. The introduction of a professional caregiver into family health teams, who assists in the task of caring for dependent elderly people in their homes for a few hours a week, freeing the family member to enjoy some free time, could contribute to alleviating the burden of CFID . This work aims to evaluate the effectiveness of a type of intervention based on material support to CFID, provided by a professional, in a region with a predominance of violence and socio-environmental vulnerability.

2. OBJECTIVES

Main goal

To evaluate the effectiveness of care provided by itinerant professional caregivers in relieving the burden of family caregivers of dependent elderly people.

Specific objectives

1) Compare the level of burden on family caregivers before and after home care provided by formal caregivers.

2) Verify the reduction of depressive symptoms in family caregivers after home care by formal caregivers.

3) Compare the perception of social support in family caregivers before and after home care provided by formal caregivers.

3. JUSTIFICATION

Community volunteer work as temporary caregivers, in order to give family caregivers a break, has been encouraged in the United States16, but this practice is not common in our country. Furthermore, there is already evidence in international studies on the effectiveness of specific interventions to reduce CFID burden in certain populations. The 10/66 Dementia Reseach Group conducts population-based research on dementia in low- and middle-income countries, and aims to provide evidence that supports the implementation of policies to improve the health and social well-being of older people and their caregivers17 . One of its programs, delivered in five sessions at home, consists of skills training to deal with the disease. The intervention, tested in Peru18 and Russia19, was effective in reducing caregiver burden after a 6-month follow-up.

In Brazil, initiatives involving the employment of formal home caregivers as part of public policies are still few in number and restricted to some capitals, an example being the Elderly Companion Program of the Health Department of the city of São Paulo, which occurs in an integrated manner to the primary care policy of that municipality20

A recent study carried out in the Manguinhos region (submitted in April 2015 to the journal Cadernos de Saúde Pública), whose objective was to analyze the prevalence of caregiver overload and factors associated with it, revealed that the group of dependent elderly people was predominantly made up of women widowed or separated, with less than four years of education, suffering from depression (60%) and cognitive decline (87.6%). In the group of caregivers, there was a predominance of middle-aged women, with a third reporting unsatisfactory social support and almost 60% showing signs of moderate to high burden. Furthermore, worrying rates of alcohol problems were identified (18%). In the analysis of factors associated with caregiver stress, it was found that the occurrence of depression and cognitive decline in the elderly considerably increased the risk of overload, with odds ratios equal to 2.59 (p= 0.005) and 3.19 (p= 0.03), respectively. In relation to caregivers, only social support remained relevant (odds ratio= 2.35; p= 0.01). When asked about the type of help they would like to get with care, most mentioned help with care.

The results of this work constituted the basis for the intervention study proposed here, whose hypothesis is that an intervention focused on freeing the caregiver to have free time on a regular basis is capable of reducing symptoms of overload and could have an impact on care. of dependent elderly people in a community with few resources for the formal care of this type of patient.

4. METHOD

Study Design: randomized controlled clinical trial.

Study Location: the study will be carried out in the Manguinhos region, a region with full coverage by the Family Health Strategy (ESF) but with extreme socio-environmental vulnerability, divided into sub-areas, where approximately 36 thousand people lived in 2011. The households, built in masonry, had an average of 2.8 inhabitants each; most had just one room; almost 60% did not have access to water from the unofficial network and more than 30% were not connected to the general sewage network. The average household income was around 630 reais per month, slightly higher than the minimum wage in force at the time. As for education, almost 50% of residents had only completed elementary school21. With a Human Development Index of 0.72622, the Manguinhos neighborhood ranked 122nd among the city's 126 neighborhoods23. In this area, initiatives to reduce violence have faced obstacles due to the actions of drug traffickers24.

Duration of the Study: the intervention period will be six months, taking as a reference previous studies with positive results in relation to overload18,19.

Study population: the 136 elderly/caregiver pairs remaining from the previous study, carried out in 2011, related to the identification of the prevalence of overload and the factors associated with it, in the Manguinhos region, whose sample consisted of elderly people, will be invited to the study. aged 60 and over of both sexes, dependent for two or more ADLs, in order to select those whose caregivers would be more prone to stress related to the act of caring, given the association between dependence and caregiver burden1. Everyone had a family caregiver responsible for supervising and caring for most activities related to the elderly.

The CFs selected to work in the research will be graduates of a training course provided by the Joaquim Venâncio Polytechnic School (EPJV), with a course load of 200 hours, in which basic knowledge about aging and care is taught, through theoretical and practical classes. Each CF will be able to care for up to six dependent elderly people, one in the morning and one in the afternoon, on Mondays, Wednesdays and Fridays. The other four will be attended only once a week, Tuesday or Thursday, in the morning or afternoon.

Interventions: Interventions will consist of:

a) Group 1: assistance by a formal caregiver for three hours, three times a week;

b) Group 2: assistance by a formal caregiver for three hours, once a week;

c) Group 3: will receive a single visit from a nurse who will provide CFID training in skills for bathing, feeding, moving, and addressing behavioral changes for 2 hours.

Study stages

CFIDs will be visited by a member of the research team at home in order to be invited to participate in the study. After acceptance, the initial interview will be carried out, where questionnaires and scales for overload25, depression26 and social support27 will be applied in the initial interview and within a month after the end of the intervention. The volunteers' responses will be recorded on a tablet-type mobile electronic equipment and sent via the 3G cell phone network, automatically to a remote database, on the server of the company hired for this purpose, where the data will be validated, processed , tabulated and made available to researchers. Following the initial interview, CFID will be randomized into three different intervention groups

The second visit will be made, either by the nurse, for skills training for two hours, or by the CF, to begin their activities. CFIDs in groups 1 and 2 will be able to delegate tasks related to the care of dependent elderly people to the CF, both for basic ADL and IADL, including monitoring activities outside the home. If transportation is needed, travel must be provided by the family member. During this period, the CFID will be able to dedicate themselves to their own tasks, including being able to be away from home. CF tasks may be modified over time, maintaining the focus on ADL and IADL. The CF's performance will be evaluated monthly by the field coordinator, through telephone contact with the CFID for which he is working and also through fortnightly meetings with field supervisors, held at EPJV.

Data collection

1) Initial interview to collect sociodemographic data and apply the following questionnaires/scales:

The. Burden Interview Scale: assesses the impact of mental and physical illnesses on informal caregivers, providing the level of emotional burden on caregivers. This scale was translated and validated in Brazil by Scazufca in 2002, consisting of 22 questions that have five possible answers, indicating how often the individual feels overwhelmed in different situations. For each alternative, the score varies from 0 (never) to 4 (almost always). Thus, estimated degrees of overload can be observed, in which: 0 – 20 indicates small overload; 21 – 40 indicates moderate overload; from 41 – 60, moderate to severe overload and 61 – 88, severe overload 25.

B. Patient Health Questionnaire-9 (PHQ-9): This test is used to screen for depression in primary care. It consists of nine questions that assess the presence of the following symptoms of depression: depressed mood, anhedonia (loss of interest or pleasure in doing things), problems with sleeping, tiredness or lack of energy, change in appetite or weight, feeling of guilt or worthlessness, problems concentrating, feeling sluggish or restless, and suicidal thoughts. The translation of the PHQ-9 into Portuguese is available online (http://www.phqscreeners.com, accessed on May 12, 2015). The frequency of each symptom in the last two weeks is evaluated on a Likert scale from 0 to 3 corresponding to the answers “none of the time”, “several days”, “more than half of the days” and “almost every day”, respectively. The questionnaire also includes a tenth question that assesses the interference of these symptoms in the performance of daily activities, such as working and studying. The cutoff for depression is ≥ 9. 26

w. Medical Outcomes Study Social Support Scale - MOS. It has 19 questions that cover the five dimensions of social support. The score is defined according to the frequency of support perceived by the individual in each of the dimensions, and can range from zero (never) to four (always). In this study, support was considered unsatisfactory when there were more than 50% of items in each dimension with answers equal to zero, one (rarely) or two (sometimes)27;

2) Final interview - the assessment will be repeated with the four instruments after six months.

Definition of outcomes (after six months of intervention):

Primary outcome: the primary outcome will be considered the reduction of at least one CFID burden level in the burden scale classification.

Secondary outcomes:

• Reduction in depression levels - improvement in depression will be considered when the score goes from nine or more to less than nine points.

• Improvement in levels of social support - an increase in social support will be considered when a category goes from unsatisfactory to satisfactory in the second assessment and this determines a statistically significant improvement in total social support.

Randomization strategy

Participants will be assigned to one of the study groups using a list of computer-generated random numbers, in blocks defined by a statistician not directly involved in the fieldwork and data analysis. The list with the random sequence designating the participation group will be used to make self-adhesive labels and the steps followed by the statistician in this process will be recorded for future reference. The labels will be placed in opaque and sealed envelopes, externally numbered with the sequence of natural numbers. Only after signing the free and informed consent form, the envelope will be opened to reveal the group in which that participant will be allocated. The identification number of the research participants will be the number of the envelope opened for him (her). The self-adhesive label with the comparison group will be pasted in the study participant's medical record. Opened envelopes will be kept for study auditing.

Strategy for reducing selection bias

Initially, an investigator will carry out initial interviews with all previously randomized pairs. At the end of the study, another investigator, blinded to the allocation of intervention groups, will reapply the instruments, with the aim of measuring the outcome.

To minimize possible losses resulting from dissatisfaction with the formal caregiver, these professionals will be exchanged upon request from the family member. Furthermore, data from individuals who leave the study due to dropout or withdrawal will be analyzed and compared with those who remain in the study.

Data analysis

The main analysis will be carried out according to intention to treat, which consists of keeping participants in the groups to which they were initially allocated. The burden reduction proportions of the intervention groups will be compared with each other.

A descriptive analysis of the distribution of CFID will be carried out in each intervention group according to

Ethical aspects

The project will be forwarded to the Research Ethics Committee (CEP) of the National School of Public Health (ENSP) of the Oswaldo Cruz Foundation (FIOCRUZ). All caregivers involved will be duly informed through the Informed Consent Form. Cases in which overload or depression are identified will be referred to the ESF, which has a multidisciplinary team.

The risks of malpractice on the part of FCs, related to the intervention, will be minimized by hiring professionals trained and supervised by members of the research team. If the family member is dissatisfied with the professional's performance, they will be replaced.

The benefits that involve participation are related to improving the health of participants who have social support provided by the CF.

5. FLOW CHART

G1

G2

G3

Family caregivers

Randomization

Initial assessment

Inervention

Final assessment after 6 months

6. EXPECTED RESULTS

It is expected that the introduction of a formal caregiver, working a few hours a week with dependent elderly people, will provide a reduction in the burden of CFID and provide the SUS with knowledge of an accessible technology for treating the burden of CFID. It is also intended to develop a project for care policy for dependent elderly people for the city of Rio de Janeiro, based on the experience and results obtained in the research.

The publication of a scientific article in an indexed journal (A1) is the last expected result.

1. EXECUTION CHRONOGRAM

| **activity** | **First year/ months** | **Second year/ months** |
| --- | --- | --- |
| **LITERATURE REVIEW** | Jun-Jul |  |
| **RANDOMIZATION/GROUP DIVISION** | Aug |  |
| **INITIAL INTERVIEW AND QUESTIONNAIRE** | Aug-Sep |  |
| INTERVENTION | Aug-Feb |  |
| FINAL ASSESSMENT |  | Feb-Mar |
| DATA ANALYSIS |  | Apr-May |
| WRITING, TRANSLATION AND ARTICLE SUBMISSION |  | Jun-Sep  Aug –Oct |
|  |  |  |

1. BUDGET

| **Product/ service** | **Quantity** | **Unitary value R$** | **Subtotal** | **Total R$** |
| --- | --- | --- | --- | --- |
| - Development of the smartphone questionnaire  -Development of study monitoring tools  -Data processing during the project period  -Rental of equipment with 3G internet and insurance  - Taxes |  |  | 9.200,00  5.100,00  1.200,00  5.400,00  3.553,00 | 24.453,00 |
| Grant for formal caregivers (6 hours/day/6 months) | 12 | 1.310,00  x 6= 7.860,00 |  | 94.320,00 |
| Transportation vouchers | 12- 20 times a month/ 6 m | 3,40 x 2 - 20 (days) x 6 (m)= | 816,00 | 9.792,00 |
| Meal ticket | 12 people- 20 mals/m/6 m | 12,00 x 20 (days) x 6 m | 240,00/ m x 12=2.880,00 | 17.280,00 |
| Grant (initial assessment) | 1 | 2.000,00/month | 2 m | 4.000,00 |
| Grant (final assessment | 1 | 2.000,00/m | 2 m | 4.000,00 |
| Grant (field coordinator) | 1 | 2.000,00/m | 7 m | 14.000,00 |
| Grant (nurse) | 1 | 2.000,00/m | 2 m | 4.000,00 |
| Translation and publication of manuscript | 1 | 8000,00 |  | 8.000,00 |
| buffet brunch to 40 people |  |  | 29,99 | 1.199,60 |
| Round-trip ticket to Brasilia | 1 |  |  | 2.040,00 |
| **TOTAL** |  |  | **183.084,60** | |

1. BIBLIOGRAPHIC REFERENCES
2. World Health Organization. World Report on Disability. In: Bank. W, ed. Geneva; 2011: 350.
3. Lima-Costa MF, Matos DL, Camargos VP, Macinko J. 10-year trends in the health of Brazilian elderly: evidence from the National Household Sample Survey (PNAD 1998, 2003, 2008). *Ciencia & saude coletiva.* Sep 2011;16(9):3689-3696.
4. Garbin CA, Sumida DH, Moimaz SA, do Prado RL, da Silva MM. Aging by the perspective of elderly caregivers. *Ciencia & saude coletiva.* Sep 2010;15(6):2941-2948.
5. Caldas CP.Aging with dependence: family needs and responsibilities. *Cadernos de Saude Publica.* May-Jun 2003;19(3):773-781.
6. Chappell NL, Funk LM. Social support, caregiving, and aging. *Canadian Journal on Aging.* Sep 2011;30(3):355-370.
7. Oliveira DC, D'Elboux MJ. National studies on family caregivers of older persons: integrative review. *Rev Bras Enferm.* 2012 Sep-Oct 2012;65(5):829-838.
8. Rospenda KM, Minich LM, Milner LA, Richman JA. Caregiver burden and alcohol use in a community sample. *Journal of Addictive Diseases.* Jul 2010;29(3):314-324.
9. Rodakowski J, Skidmore ER, Rogers JC, Schulz R. Role of social support in predicting caregiver burden. *Archives of physical medicine and rehabilitation.* Dec 2012;93(12):2229-2236.
10. Viana MC, Gruber MJ, Shahly V, Alhamzawi A, Alonso J, Andrade LH et al. Family burden related to mental and physical disorders in the world: results from the WHO World Mental Health surveys. *Rev Bras Psiquiatr.* 2013;35(2):115-125.
11. Álida Rosária Silva Ferreira; Laura Rodriguez Wong. Perspectivas da oferta de cuidadores informais da população idosa, Brasil 2000-2015. <http://www.abep.nepo.unicamp.br/encontro2008/docsPDF/ABEP2008_1624.pdf>) acesso em 15-6-13
12. Shye D, Mullooly JP, Freeborn DK, Pope CR. Gender differences in the relationship between social network support and mortality: a longitudinal study of an elderly cohort. *Social Science & Medicine.* Oct 1995;41(7):935-947.
13. Sherbourne CD, Stewart AL. The MOS social support survey. *Social science & medicine.* 1991;32(6):705-714.
14. Guedea MTD, Damacena, F. A., Carbajal, M. M. M., Marcobich, P. O., Hernández, G. A., Lizárraga, L. V.Flores, E. I. . Social support needs of mexican elders family caregivers. *Psicologia & Sociedade.* 2009;21 (2):7.
15. Robinson-Whelen S, Tada Y, MacCallum RC, McGuire L, Kiecolt-Glaser JK. Long-term caregiving: what happens when it ends? J Abnorm Psychol. 2001 Nov;110(4):573-84.
16. Chien LY, Chu H, Jong-Long G, Yuan-Mei L, Lu-I C, Chiung-Hua C and Kuei-Ru C. Caregiver support groups in patients with dementia: a meta-analysis. Int J Geriatr Psychiatry 2011; 26: 1089–1098.
17. Department of Health and Human Services. Administration for Community Living (ACL). http://www.acl.gov/NewsRoom/Observances/WEAAD/Get-Involved/Take-Action.aspx- acesso em 23 jan 2015.
18. Prince M. Dementia Research Group. 2004. Care arrangements for people with dementia in developing countries. Int J Geriatr Psychiatry 19: 170–177.
19. Guerra, Mariella; Ferri, Cleusa; Fonseca, Magaly; Banerjee, Sube; Prince, Martin. Helping carers to care: the 10/66 Dementia Research Group`s randomized control trial of a caregiver intervention in Peru. Peru, 2010. Revista Brasileira de Psiquiatria, 33(1)
20. Gavrilova, I. Svetlana; Ferri, P. Cleusa; Mikhaylova, Natalya; Sokolova, Olga; Banerjee, Sube; Prince, Martin. Helping cares to care – The 10//66 Dementia Research Group`s randomized control trial of a caregiver intervention in Russia, 2009. Rússia. International Journal of Geriatric Psychiatry, 24, 347-354
21. Berzins MAVS et al. Programa “acompanhante de idosos”. Bol Instit Saude. 2009;(47):53-5
22. Schütz G, Pivetta F, Engstron E. Contexto do TEIAS-Escola Manguinhos. In: MAP C, F P, eds. *The integrated territory of health care in Manguinhos: we are all apprentices!* Rio de Janeiro: National Public Health School/ Oswaldo Cruz Foundation; 2012: 183.
23. WikiRio [Internet]: Rio de Janeiro City HDI. [acesso em 10 de março de 2015]. Disponível em <http://www.wikirio.com.br/IDH_dos_bairros_da_cidade_do_Rio_de_Janeiro>
24. Laboratorio Territorial de Manguinhos [Internet]. Territorio e cidadania. *Escola Nacional de Saude Publica*. [acesso em 12 de janeiro de 2015]. Disponível em http://www.conhecendomanguinhos.fiocruz.br/.
25. de Souza ER, Ribeiro AP, Atie S, de Souza AC, Marques Cda C. The net for protection to the elderly of Rio de Janeiro: a right to be conquered. *Ciencia & saude coletiva.* Jul-Aug 2008;13(4):1153-1163.
26. Scazufca M, Menezes PR, Almeida OP. Caregiver burden in an elderly population with depression in Sao Paulo, Brazil. *Social Psychiatry and Psychiatric Epidemiology.* Sep 2002;37(9):416-422.
27. Santos I,Tavares BF, Munhoz TN, Almeida LSP, Silva NTB, Tams BD et al.Sensibilidade e especificidade do Patient Health Questionnaire-9 (PHQ-9) entre adultos da população geral. Cad. Saúde Pública [online]. 2013, vol.29, n.8, pp. 1533-1543.
28. Cunha JA. Manual da versão em português das Escalas Beck. São Paulo: Casa do Psicólogo, 2001.
29. Griep RH, Chor D, Faerstein E, Werneck GL, Lopes CS. Construct validity of the Medical Outcomes Study's social support scale adapted to Portuguese in the Pro-Saude Study. *Cadernos de Saude Publica.* 2005;21(3):703-714.

10. ATTACHMENTS

1. ANNEX I

Research protocol

**Family caregiver**

1. Marital Status: ( ) married (lives in a union) ( ) separated ( ) widowed ( ) single

2. How old are you?_________ Date of birth______________

3. What is your education level (in years of study)________

4. Do you live with the elderly person?

5. How many hours do you dedicate daily to caring for the elderly?________

**6. Caregiver Burden Inventory – Zarit: total score______________**

| INVENTÁRIO DE SOBRECARGA DE ZARIT |  | | | | |
| --- | --- | --- | --- | --- | --- |
|  | 0 | 1 | 2 | 3 | 4 |
|  | NUNCA | RARAMENTE | ALGUMAS VEZES | MUITO FREQUENTE | QUASE SEMPRE |
| 1. Do you feel that S* asks for more help than he/she needs? | 0. | 1. | 2. | 3. | 4. |
| 2. Do you feel that because of the time you spend with S*, you do not have enough time for yourself? | 0. | 1. | 2. | 3. | 4. |
| 3. Do you feel stressed between taking care of S* and your other responsibilities with family and work? | 0. | 1. | 2. | 3. | 4. |
| 4. Do you feel embarrassed by S*’s behavior? | 0. | 1. | 2. | 3. | 4. |
| 5. Do you feel irritated when S* is around? | 0. | 1. | 2. | 3. | 4. |
| 6. Do you feel that S* negatively affects your relationships with other family members or friends? | 0. | 1. | 2. | 3. | 4. |
| 7. Do you feel afraid for S*’s future? | 0. | 1. | 2. | 3. | 4. |
| 8. Do you feel that S* depends on you? | 0. | 1. | 2. | 3. | 4. |
| 9. Do you feel tense when S* is around? | 0. | 1. | 2. | 3. | 4. |
| 10. Do you feel that your health has been affected because of your involvement with S*? | 0. | 1. | 2. | 3. | 4. |
| 11. Do you feel that you do not have as much privacy as you would like, because of S*? | 0. | 1. | 2. | 3. | 4. |
| 12. Do you feel that your social life has suffered because you are looking after S*? | 0. | 1. | 2. | 3. | 4. |
| 13. Do you not feel comfortable having visitors at home because of S*? | 0. | 1. | 2. | 3. | 4. |
| 14. Do you feel that S* expects you to take care of him/her, as if you are the only person he/she can depend on? | 0. | 1. | 2. | 3. | 4. |
| 15. Do you feel that he does not have enough money to take care of S*, in addition to his other expenses? | 0. | 1. | 2. | 3. | 4. |
| 16. Does Mr/Mrs feel that he will be unable to look after S* much longer? | 0. | 1. | 2. | 3. | 4. |
| 17. Do you feel that you have lost control of your life since S*'s illness? | 0. | 1. | 2. | 3. | 4. |
| 18. Would you like to just let someone else take care of S*? | 0. | 1. | 2. | 3. | 4. |
| 19. Do you feel in doubt about what to do for S*? | 0. | 1. | 2. | 3. | 4. |
| 20. Does Mr/Mrs feel he should be doing more for S*? | 0. | 1. | 2. | 3. | 4. |
| 21. Does Mr/Mrs feel he could take better care of S*? | 0. | 1. | 2. | 3. | 4. |
| 22. In general, how burdened do you feel by taking care of S*? | 0. | 1. | 2. | 3. | 4. |
| TOTAL OF POINTS: |  |  |  |  |  |

**7. Social support: total______________**

| Social support | | | | | |
| --- | --- | --- | --- | --- | --- |
| “If you need it, how often can you count on someone... | | | | | |
|  | 1-never | 2-rarely | 3-sometimes | 4-almost always | 5-always |
| Material |  |  |  |  |  |
| 1. What will help you if you stay in bed? |  |  |  |  |  |
| 1. To take you to the doctor |  |  |  |  |  |
| 1. To help you with daily tasks if you get sick? |  |  |  |  |  |
| 1. To prepare your meals if you can't prepare them |  |  |  |  |  |
| Emotional |  |  |  |  |  |
| 1. To listen to you when you need to speak? |  |  |  |  |  |
| 1. Who can you trust to talk about you or your problems? |  |  |  |  |  |
| 1. To share your most intimate worries and fears? |  |  |  |  |  |
| 1. Who understands your problems? |  |  |  |  |  |
| Affective |  |  |  |  |  |
| 1. SShows love and affection for you? |  |  |  |  |  |
| 1. 1Gives you a hug? |  |  |  |  |  |
| 1. 1That you love and that makes you feel wanted? |  |  |  |  |  |
| Information |  |  |  |  |  |
| 1. 1To give good advice in crisis situations? |  |  |  |  |  |
| 1. 1To provide information that helps you understand a certain situation? |  |  |  |  |  |
| 1. Who do you really want advice from? |  |  |  |  |  |
| 1. 1To give suggestions on how to deal with a personal problem? |  |  |  |  |  |
| Positive social interaction |  |  |  |  |  |
| 1. Who to do nice things with? |  |  |  |  |  |
| 1. Who to take your mind off? |  |  |  |  |  |
| 1. Who to relax with? |  |  |  |  |  |
| 1. To have fun together? |  |  |  |  |  |

**8. PHQ-9: total score_____________**

**Portuguese version of the Patient Health Questionnaire-9 (PHQ-9)**

**NOW LET'S TALK ABOUT HOW YOU HAVE BEEN FEELING THE LAST TWO WEEKS.**

**1) In the last two weeks, how many days did you have little interest or little pleasure in doing things?**

**(0) No day**

**(1) Less than a week**

**(2) A week or more**

**(3) Almost every day**

**2) In the last two weeks, how many days did you feel down, depressed or without perspective?**

**(0) No day**

**(1) Less than a week**

**(2) A week or more**

**(3) Almost every day**

**3) In the last two weeks, how many days did you have difficulty falling asleep or staying asleep or did you sleep more than usual?**

**(0) No day**

**(1) Less than a week**

**(2) A week or more**

**(3) Almost every day**

**4) In the last two weeks, how many days did you feel tired or had little energy?**

**(0) No day**

**(1) Less than a week**

**(2) A week or more**

**(3) Almost every day**

**5) In the last two weeks, how many days did you have a lack of appetite or eat too much?**

**(0) No day**

**(1) Less than a week**

**(2) A week or more** (3) Quase todos os dias

6) In the last two weeks, how many days have you felt bad about yourself or felt that you are a failure or that you have let your family or yourself down?

(0) No day

(1) Less than a week

(2) A week or more

(3) Almost every day

7) In the last two weeks, how many days did you have difficulty concentrating on things (such as reading the newspaper or watching television)?

(0) No day

(1) Less than a week

(2) A week or more

(3) Almost every day

8) In the last two weeks, how many days were you slow to move or speak (to the point where other people noticed), or on the contrary, were you so agitated that you kept walking at a side to side more than usual?

(0) No day

(1) Less than a week

(2) A week or more

(3) Almost every day

9) In the last two weeks, how many days did you think about hurting yourself in some way or that it would be better to be dead?

(0) No day

(1) Less than a week

(2) A week or more

(3) Almost every day

10) Considering the last two weeks, have the previous symptoms caused you any type of difficulty in working or studying or taking care of things at home or interacting with people?

(0) No difficulties

(1) Little difficulty

(2) Much difficulty

(3) Extreme difficulty

ANNEX II page 1/2

**FREE AND INFORMED CONSENT FORM (ICF) - CAREGIVER**

**Effectiveness of care provided by itinerant professional caregivers in reducing the burden on family caregivers of dependent elderly people**

**Dear Caregiver:**

**You are being invited to participate in the research Effectiveness of care provided by itinerant professional caregivers in reducing the burden on family caregivers of dependent elderly people. This research is being coordinated by Valéria Lino and Gisele O'Dwyer, researchers at the Sergio Arouca National School of Public Health of the Oswaldo Cruz Foundation (ENSP/FIOCRUZ).**

**The objective of the study is to verify whether the participation of a professional caregiver for a few hours a week in caring for the elderly, or the training of the family caregiver by a nurse, are capable of alleviating the burden on family caregivers of dependent elderly people.**

**The invitation to participate is due to the fact that you are a caregiver for a dependent elderly person. Your participation is voluntary, that is, it is not mandatory and you can decide whether or not to participate, as well as withdraw your participation at any time. You will not be penalized in any way if you decide not to consent to your participation, or to withdraw from it. However, it is very important for carrying out the research. If you suffer any harm resulting from your participation in the research, you will be entitled to full assistance for treatment, in addition to compensation, as per items III.2.0, IV.4.c, V.3, V.5 and V6 of resolution CNS466/12.**

**Your participation will consist of answering questions at the beginning and end of the research, after six months. The questionnaire application time is 20 minutes. In addition, a nurse can come and train you in your home for 2 hours on appropriate ways to care for dependent elderly people, or you will receive help from a professional caregiver for a few hours, a few days a week.**

**You may benefit from receiving training to care for your elderly relative, as the knowledge gained during this time will help you provide better care. If you join the group that will receive a caregiver for some time, the time off you will have during these times can alleviate your feelings of stress and tiredness related to caregiving. Furthermore, as this research aims to test a form of care for dependent elderly people at home, we believe that it can be incorporated into the SUS in the future, if it demonstrates benefits for the public health system.**

**______________ Researcher Rubric**

**_____________ Participant Rubric**

pag 2/2

The information you provide will be confidential and your data will be stored in a database along with those of the other participants, without identification by name, only by numbers. Even so, there is a possibility that you will be recognized, as few family caregivers will participate in this research.

At any time, during the research, or later, you may request information from the researcher about your participation and/or the research, which can be done through the contact methods explained in this Term.

The risks possibly related to this research may be related to the care provided by professional caregivers to your elderly person. However, they will be trained by Fiocruz teams and monitored throughout the research.

At the end of the research, all material will be kept on file for at least 5 years, in accordance with CNS Resolution 466/12 and CEP/ENSP guidelines.

If you have any doubts regarding the ethical conduct of the study, contact the ENSP Research Ethics Committee (CEP), which aims to defend the interests of research participants and ensure that the research takes place within ethical standards. The CEP monitors and monitors the research, so that it respects the ethical principles of protecting human rights, dignity, autonomy, confidentiality, privacy and non-maleficence.

This term is written in two copies, one from you and the other from the researcher.

Rio de Janeiro, _____ of ____________, 2015.

__________________________________________________

Signature of the Principal Investigator

Address: Germano Sinval Farias School Health Center, Sergio Arouca National School of Public Health/ FIOCRUZ, Rua Leopoldo Bulhões, 1480 – Ground Floor - Manguinhos - Rio de Janeiro – RJ - CEP: 21041-210

I declare that I understand the objectives and conditions of my participation in the research and I agree to participate.

_________________________________________

Participant signature

CEP/ENSP. Tel and Fax - (0XX) 21- 25982863; e-mail: cep@ensp.fiocruz.br; http://www.ensp.fiocruz.br/eticaANEXO III pag 1/2

**FREE AND INFORMED CONSENT FORM (ICF) - ELDERLY**

**Effectiveness of care provided by itinerant professional caregivers in reducing the burden on family caregivers of dependent elderly people**

**Dear Sir/Madam:**

**You are being invited to participate in the research Effectiveness of care provided by itinerant professional caregivers in reducing the burden on family caregivers of dependent elderly people.**

**The research is being coordinated by Valéria Lino and Gisele O'Dwyer, researchers at the Sergio Arouca National School of Public Health of the Oswaldo Cruz Foundation (ENSP/FIOCRUZ).**

**The objective of the study is to verify whether the participation of a professional caregiver, for a few hours a week, helping with their care, or the training of their family caregiver by a nurse, are capable of alleviating the burden on family caregivers of elderly people who need of help for everyday life.**

**The invitation to participate is due to the fact that you need some help with your day-to-day tasks at home. Your participation is voluntary, that is, it is not mandatory and you can decide whether or not to participate, as well as withdraw your participation at any time. You will not be penalized in any way if you decide not to consent to your participation, or to withdraw from it. However, it is very important for carrying out the research. However, if you suffer any harm resulting from your participation in the research, you will be entitled to full assistance for treatment, in addition to compensation, as per items III.2.0, IV.4.c, V.3, V.5 and V6 of resolution CNS466/12, which regulates research in our country.**

**The information you provide will be confidential and your data will be stored in a database along with those of the other participants, without identification by name, only by numbers. Even so, there is a possibility that you will be recognized, as few family caregivers will participate in this research.**

**Your participation will consist of accepting help from a professional caregiver, whose role will depend on what is agreed with you and your family caregiver, or your family caregiver will be trained by a nurse on appropriate techniques for caring for you, such as: removing bed, bathing, feeding, adequate exercise, etc.**.

**______________ Researcher Rubric**

**_____________ Participant Rubric**

pag2/2

You may benefit from receiving care from a professional during your time in study. Likewise, your relative may have a little time off while you are with the caregiver. Or, if you join the group whose caregiver will be trained by a nurse, you will benefit from the learning, as it will provide better care.

Furthermore, as this research aims to test a form of care for dependent elderly people at home, we believe that it can be incorporated into the SUS in the future, if it demonstrates benefits for the public health system.

At any time, during the research, or later, you may request information from the researcher about your participation and/or the research, which can be done through the contact methods explained in this Term.

Risks possibly related to this research may be related to the care provided to you by professional caregivers. However, they will be trained by Fiocruz teams and monitored throughout the research.

At the end of the research, all material will be kept on file for at least 5 years, in accordance with CNS Resolution 466/12 and CEP/ENSP guidelines.

If you have any doubts regarding the ethical conduct of the study, contact the ENSP Research Ethics Committee (CEP), which aims to defend the interests of research participants and ensure that the research takes place within ethical standards. The CEP monitors and monitors the research, so that it respects the ethical principles of protecting human rights, dignity, autonomy, confidentiality, privacy and non-maleficence.

This term is written in two copies, one from you and the other from the researcher.

Rio de Janeiro, _____ of ____________, 2015

__________________________________________________

Signature of the Principal Investigator

Address: Germano Sinval Farias School Health Center, Sergio Arouca National School of Public Health/ FIOCRUZ, Rua Leopoldo Bulhões, 1480 – Ground Floor - Manguinhos - Rio de Janeiro – RJ - CEP: 21041-210

I declare that I understand the objectives and conditions of my participation in the research and I agree to participate.

_________________________________________

Participant signature

CEP/ENSP. Tel and Fax - (0XX) 21- 25982863; e-mail: cep@ensp.fiocruz.br; http://www.ensp.fiocruz.br/etica
