# Supplementary material for: Effectiveness of care provided by an itinerant community caregiver in reducing the burden and violence of family caregivers of impaired elderly in Rio de Janeiro, Brazil: A randomized clinical trial
Source: PLoS One. 2024 Dec 5;19(12):e0309712. doi: 10.1371/journal.pone.0309712 (PMC11620672; doi:10.1371/journal.pone.0309712)
Supplement: S1 Fig — (PDF) [file pone.0309712.s003.pdf]

NATIONAL SCHOOL OF HEALTH SERGIO AROUCA PUBLIC - ENSP/FIOCRUZ

Researcher: Valéria Teresa Saraiva Lino

Search Title: Effectiveness of care provided by itinerant professional caregivers in reducing overload of family caregivers of dependent elderly people: randomized clinical trial

Proposing Institution: OSWALDO CRUZ FOUNDATION

Version:3

CAAE: 45595215.4.0000.5240

Main Sponsor: Own Financing

Thematic Area:

RESEARCH PROJECT DATA

Opinion Number: 1,356,166

This is a response to the consolidated opinion of CEP/ENSP number 1,321,797, of November 13, 2015. The research project in question obtained the consolidated opinion of CEP/ENSP number 1,152,711, of July 17, 2015. It is Valéria Teresa Saraiva Lino's own project, with the following research team: Daniel Groisman; Fabiana Lima Rajão; Germana Périssé de Abreu; Gisele O'Dwyer de Oliveira; Ines Echenique Mattos; Idenalva Silva de Lima; Luiz Antonio Bastos Camacho; Nádia Cristina Pinheiro Rodrigues and Monica Kramer de Noronha Andrade. The project budget is R$189,044.60, reported as own financing. According to the researcher, "The project will participate in a competition for financing by Fiocruz - "Public Policies and Health Care Models", whose notice was launched in April 2015, with financing of up to 200 thousand reais per project. If it is not included, no will be realized".

"Summary:

Approximately 200 million individuals around the world have functional dependence, requiring assistance to perform daily activities, situation with an increasing trend due to population aging and the increase in the prevalence of chronic diseases. But despite knowledge of rising levels of disability, we are still not capable of measuring the impact on the health system, both of dependence and of the need of caregivers for dependent elderly people. Recognizing the role of the caregiver and carrying out estimates about this population in our environment constitute a need to be considered in the public policy planning. This study aims to evaluate the effectiveness of care provided by itinerant professional caregiver to alleviate the burden of family caregivers of elderly people dependent on through a randomized controlled clinical trial lasting six months. Formal caregivers will be allocated to homes for dependent elderly people who have family caregivers for some hours per week. Overload, depression and social support scales will be applied before and after the end of the study. The primary outcome will be the reduction of at least one level of family caregiver burden on the overload scale rating. Secondary outcomes will be the reduction of depression and improved social support. The main analysis will be carried out according to intention to treat, which consists of keep participants in the groups to which

they were initially allocated. The proportions of reduction of burden of the intervention groups will be compared with each other.

"Proposed Methodology:

Study Design: randomized controlled clinical trial.

Study Location: the study will be carried out in the Manguinhos region, a region with full coverage by Family Health Strategy (ESF) but with extreme socio-environmental vulnerability, divided into subareas, where approximately 36 thousand people lived in 2011. The homes, made of masonry, they had an average of 2.8 inhabitants each; most had just one room; almost 60% do not they had access to water from an unofficial network and more than 30% were not connected to the general sewage network. A average household income was around 630 reais per month (U$100) , slightly higher than the minimum wage in force at the time. Regarding education, almost 50% of residents had only completed secondary education. With a Human Development Index of 0.72622, the Manguinhos neighborhood occupied the 122nd place among the city's 126 neighborhoods. In this area, initiatives to reduce violence have faced obstacles due to the actions of drug traffickers.

Duration of the Study: the intervention period will be six months, taking previous studies as a reference with positive results in relation to overload.

Study population: the remaining 136 elderly/caregiver pairs will be invited to the study of the previous study, carried out in 2011, related to the identification of the prevalence of overload and factors associated with it, in the Manguinhos region, whose sample consisted of elderly people aged 60 and more of both sexes, dependent for two or more ADL, in order to select those whose caregivers would be more prone to stress related to the act of caring, given the association between dependence and burden on caregivers1. Everyone had a family caregiver responsible for their supervision and care of most activities related to the elderly. The CFs selected for performance in the research will be graduates of a training course provided by the Escola Politecnica Joaquim Venâncio (EPJV), with a workload of 200 hours, in which basic knowledge about aging and care, through theoretical and practical classes. Each CF can care for up to six elderly people dependents, one in the morning and one in the afternoon, on Mondays, Wednesdays and Fridays. The other four will be attended only once a week, Tuesday or Thursday, in the morning or afternoon.

Interventions will consist of:

a) Group 1: assistance by a formal caregiver for three hours, three times a week;

b) Group 2: assistance by a formal caregiver for three hours, once a week;

c) Group 3: will receive a single visit from a nurse who will promote CFID training in skills for bathing, feeding, moving, addressing behavior changes during 2 hours.

Study stages

The CFID will be visited by a member of the research team at home in order to be invited to participate in the study. After acceptance, the initial interview will be held, where they will be applied questionnaires and scales for overload25, depression26 and social support27 in the initial interview and in the period one month after the end of the intervention. Volunteers' responses will be recorded in a mobile electronic equipment of the Tablet type and sent via the 3G cell phone network, in a way automatically to a remote database, on the server of the

company contracted for this purpose, where the Data will be validated, processed, tabulated and made available to researchers. After initial interview, CFID will be randomized into three different intervention groups The second visit will be done, either by the nurse, for skills training for two hours, or by the CF, to start of its activities. CFIDs in groups 1 and 2 may delegate tasks related to the care of the patient to the CF dependent elderly person, both for basic ADL and for IADL, including support in activities outside the home.

Inclusion Criteria:

Caregivers of dependent elderly people who have participated in the previous study to evaluate the prevalence of overload in caregivers in the Manguinhos region". Exclusion Criteria: "caregivers of elderly people with only one dependency for activities of daily living". Data Analysis Methodology: "The main analysis will be carried out according to intention to treat, which consists of keeping participants in the groups to which they were initially allocated. The proportions of burden reduction of the intervention groups will be compared with each other. Descriptive analysis will be carried out of the distribution of CFID in each intervention group according to gender, age group, education, depression, social support, self-perceived health and burden. Proportions of burden reduction in each group, the difference between proportions, as well as the 95% CI will be calculated and the p-value of the difference between the proportions. Multivariate negative binomial and ordinal logistic regression models with proportional odds of the cumulative type will be used to evaluate the relationship between demographic characteristics, social support, depression and self-perceived health and caregiver burden. Graphical models will be used to supplement the analysis. Data entry and analysis procedures will be carried out using the public domain programs R-project 3.1.2.

Sample size in Brazil- 138.

Objectives

To evaluate the effectiveness of the care provided by an itinerant professional caregiver in alleviating the overload of family caregivers of dependent elderly people.

Secondary Objective:

1) Compare the level of burden on family caregivers before and after home care by formal caregivers.

2) Verify the reduction of depressive symptoms in family caregivers after home care by

formal caregivers.

3) Compare the perception of social support in family caregivers before and after home care

by formal caregivers."

Assessment of risks and benefits

The researcher describes the risks and benefits as:

Risks:

The risks of malpractice on the part of CFs, related to the intervention, will be minimized by hiring of professionals trained and supervised by members of the research team. If there is dissatisfaction by the family member or elderly person with the professional's work, this will be

changed. The risks involved elderly people may result from the care provided by the professional caregiver. However, this will be monitored regularly throughout the study.

Benefits:

The benefits that involve participation are related to improving the health of participants who have social support provided by the CF, or the acquisition of knowledge for better care for the dependent elderly. The acquisition of knowledge in relation to a new technology for the SUS could incur collective benefit for society and public health."

Comments and Considerations about the Research: none

It presented all the necessary terms for ethical assessment.

Considerations about Mandatory Submission Terms: Presented all necessary terms for ethical assessment.

Recommendations: Project approved

Final considerations:

In compliance with subitem II.19 of CNS Resolution No. 466/2012, it is up to the researcher responsible for present study to prepare and present a final report "[...] after the end of the research, totaling its results. The report must be sent to the CEP by Plataforma Brasil in the form of a "notification". The report model that must be followed is available at www.ensp.fiocruz.br/etica.

Any need for modification in the course of the project must be submitted to the CEP for consideration, as an amendment. You must wait for a favorable opinion from the CEP before making the change.

Justify in detail, before the CEP, if there is interruption of the project or non-publication of the results.

Rio de Janeiro, December 08, 2015

Carla Lourenço Tavares de Andrade

(Coordinator)
